# Metabarcoding and Metabolome Analyses Reveal Mechanisms of *Leymus chinensis* Growth Promotion by Fairy Ring of *Leucocalocybe mongolica*

**DOI:** 10.3390/jof8090944

**Published:** 2022-09-08

**Authors:** Mingzheng Duan, Jia Lu, Wenjing Yang, Meiling Lu, Jibin Wang, Suli Li, Yiting Chen, Lihua Hu, Lingqiang Wang

**Affiliations:** State Key Laboratory for Conservation and Utilization of Subtropical Agro-Bioresources, College of Agriculture, Guangxi University, 100 Daxue Rd., Nanning 530004, China

**Keywords:** fairy ring, *Leucocalocybe mongolica*, *Leymus chinensis*, 16S rRNA, ITS, UPLC-ESI-MS/MS, leaves, roots

## Abstract

Fairy rings are a unique ecological phenomenon caused by the growth of the fungal mycelium in the soil. Fairy rings formed by *Leucocalocybe mongolica* (LM) are generally distributed in the Mongolian Plateau, where they promote plant growth without fertilization and alleviate fertilizer use. We previously investigated the soil factors regulating growth promotion in a fairy ring ecosystem; however, the aspects of the plant (*Leymus chinensis*, LC) that promote growth have not been explored. Therefore, the present study investigated the endophyte diversity and metabolome of LC in an LM fairy ring ecosystem. We analyzed the leaf and root samples of LC from the DARK (FR) and OUT (CK) zones. The fairy rings significantly improved the fungal diversity of roots and leaves and the bacterial diversity of leaves in the FR zone. *Ralstonia* was the dominant bacteria detected in the LC leaves. In addition, *Marasmius*, another fairy ring fungal genus, was also detected with a high abundance in the roots of the FR zone. Furthermore, widely targeted metabolome analysis combined with KEGG annotation identified 1011 novel metabolites from the leaves and roots of LC and seven pathways significantly regulated by the fairy ring in the FR zone. The fairy ring ecosystem significantly downregulated the flavonoid metabolism in the leaves and roots of LC. The correlation analysis found *Ralstonia* is a potential regulatory factor of flavonoid biosynthesis in LC. In addition, salicylic acid and jasmonic acid were found upregulated in the leaves, probably related to *Marasmius* enrichment. Thus, the study details plant factors associated with enhanced growth in an LM fairy ring ecosystem. These findings lay a theoretical foundation for developing the fairy ring ecosystem in an agricultural system.

## 1. Introduction

Excessive fertilization is a major agricultural problem, which inhibits crop productivity improvement, pollutes the environment, and threatens soil biodiversity, especially in China and other developing countries [1,2,3,4]. Therefore, there is an urgent need to identify measures to efficiently use soil nutrients, balance food production, and achieve environmental goals.

The fairy ring is a unique fungal growth phenomenon usually found in grasslands and formed by particular soil-dwelling fungi, such as *Marasmius oreades* [5] and *Lepista sordida* [6]. The phenomenon can be divided into three types based on their influence on plant growth [7]. Among them, the Type I fairy ring has strong plant growth-promoting capacity and strong development potential, as observed in many areas of the world, including Europe [8] and America [9].

This complex ecological phenomenon is regulated by microbial diversity, fungal metabolites, soil properties, and plant–soil interaction [8,10,11]. Studies have described the formation of fairy rings [6,7,8,10,12,13,14]. Among different kinds of fairy ring fungi, *Leucocalocybe mongolica* (LM), an edible mushroom, is found distributed in the Inner Mongolia steppe region of China; it can significantly increase pasture (*Leymus chinensis*; LC) production without fertilization. LM fairy rings have DARK, DEAD, and OUT zones [10], with plant growth promotion mainly concentrated in the DARK zone [10]. The leaf color changes to dark green in this zone, and biomass increases compared with the OUT zone. Meanwhile, the DEAD zone, where the fungal mycelium grows in the soil, shows plant growth inhibition; consequently, the zone presents a bare soil surface. Studies have reported that the fairy ring fungi produce growth-promoting chemicals, improve soil nutrition and chemical properties (metal ions and cellulases), and increase soil metabolites [11,12,15,16,17]. However, the changes in the plants underlying the fairy ring-mediated growth promotion are unknown. Understanding these complex mechanisms will help us in artificially developing the ecological landscape and manage the problem of excessive fertilization. Recently, we investigated the soil ecology of the LM fairy rings [18]; however, the response and changes in the plants (LC) remain unknown.

Fairy rings have been correlated with changes in the microbial niche; they are known to regulate plant and soil metagenomic diversity [8,10]. Therefore, endophytes may be involved in fairy ring-mediated plant growth promotion. Typically, endophytes contribute to nutrient uptake and to improve the growth of host plants [19,20,21]. Endophytes have specifically been associated with LC plant growth [22,23,24]; therefore, enhanced endophytes may promote the growth of LC in fairy rings. Moreover, endophytes may influence host metabolism to improve disease resistance and promote growth [20,25]. Hence, fairy ring-mediated plant growth promotion may also pass through the regulation of metabolism. For example, studies have shown that metabolites such as salicylic acid and GA3 promote LC seed germination [26,27]. We previously investigated the chemical components, metabolome, and microbes of the soil ecology of fairy rings and identified numerous factors related to plant growth promotion [18]. The study found that LM can enrich soil metal ions (Fe, Mn, Zn, Cu), synthesize extra cellulase, transform soil nutrients, and regulate soil metabolites to promote the growth of LC in the DARK zone [18]. However, the corresponding plant (LC) factors have not been explored.

Therefore, the present study investigated the changes in LC with enhanced growth in the same LM fairy ring ecosystem. We analyzed the diversity of endophytes (bacteria and fungi) and metabolites in the leaves and roots of LC from the DARK and OUT zones in the LM fairy ring. We employed widely targeted metabolomics based on ultra-high-performance liquid chromatography–electrospray ionization–tandem quadrupole mass spectrometry (UPLC–ESI–MS/MS) [28,29] to analyze the metabolic differences in LC and elucidate the mechanisms underlying the growth-promoting characteristics of LM fairy rings. Finally, we propose factors associated with fairy ring-mediated plant growth promotion based on previous and current findings. 

## 2. Materials and Methods

### 2.1. Materials

**Figure 1:**
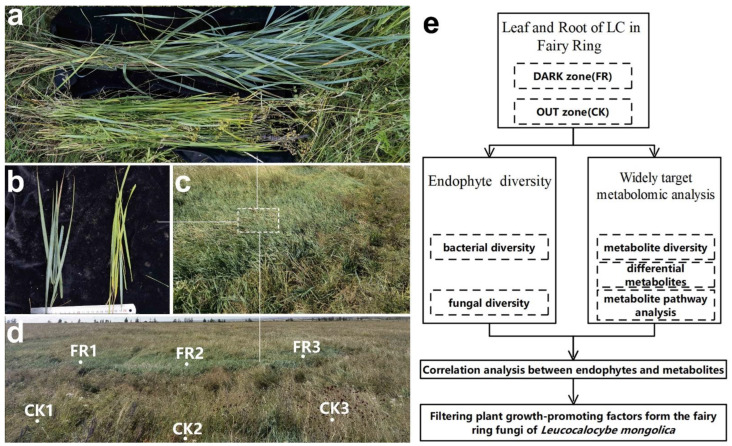
Apparent morphology and sampling spots of fairy rings and the technical route of this study. (**a**) Fairy ring landscape and sampling spots; FR1–3 represent the sampling spots in the FR zone, while CK1–3 represent the sampling spots in the CK zone. Comparison of (**b**) plant height and (**c**) leaf color between the FR (left) and CK (right) zones. (**d**) Lush vegetation inside the FR zone. (**e**) A flowchart showing the technical route of this study.

The LC leaves and roots were collected from one LM fairy ring landscape with remarkable plant growth-promoting properties located in a prairie of Baorixile Town (Hulun Buir City, Inner Mongolia autonomous region, China; 49°72′ N, 119°54′ E; an elevation of 694 m); the fairy ring analyzed in this study was the same as that in the previous study [18]. This landscape was selected as plant height (Figure 1a), leaf color (Figure 1b), and plant density and biomass (Figure 1c) were significantly higher in the DARK as compared to the OUT zones. Three sampling points were selected in the shape of an arc from the DARK (FR) and the OUT (CK) zones separately [10], as shown in Figure 1d. The samples CK1–3 and FR1–3 were used for all subsequent analyses (Figure 1d). Approximately 5 g of healthy leaf (without disease) and root samples were collected from each spot; each root sample was washed thrice with sterile water, incubated in anhydrous ethanol for 10 min, and rinsed thrice with 75% alcohol and thrice with sterile water. These leaf and root samples were immediately frozen in liquid nitrogen and stored at −80 °C for further analysis.

### 2.2. Methods

We assessed the diversity of endophytic bacteria and fungi in the leaf and root samples obtained from the FR and CK zones using high-throughput sequencing technology (Figure 1). Meanwhile, a widely targeted metabolome analysis was performed based on UHPLC–ESI–MS/MS technology to identify the metabolites and their differential expression in the leaf and root samples. Then, we tested the correlation between the identified marker endophytes and metabolites and identified the factors responsible for the growth-promoting ability of LM fairy rings.

#### 2.2.1. Metabarcoding Survey

Total DNA was extracted from the leaf and root samples using the CTAB method [30]. Then, the 16S rRNA V5–V7 region was amplified with the 799F (AACMGGATTAGATACCCKG) and 1193R (ACGTCATCCCCACCTTCC) primers for bacteria [31], and the ITS 1–2 region with the ITS1-F (CTTGGTCATTTAGAGGAAGTAA) and ITS2 (GCTGCGTTCTTCATCGATGC) primers for fungi [32]. All PCR reaction analyses were carried out with 1× Phusion^®^ High-Fidelity PCR Master Mix (New England Biolabs, Ipswic, MA, USA), 0.2 µM of forward and reverse primers, and about 10 ng template DNA. Thermal cycling consisted of initial denaturation at 98 °C for 1 min, followed by 30 cycles of denaturation at 98 °C for 10 s, annealing at 50 °C for 30 s, and elongation at 72 °C for 30 s, and finally 72 °C for 5 min. Next, sequencing libraries were generated using TruSeq^®^ DNA PCR-Free Sample Preparation Kit (Illumina, San Diego, CA, USA) following the manufacturer’s recommendations and index codes were added. The library quality was assessed on the Qubit@ 2.0 Fluorometer (Thermo Scientific, Waltham, MA, USA) and Agilent Bioanalyzer 2100 systems. Lastly, the purified amplicons were pooled in equimolar ratios and paired-end sequenced (PE250) on an Illumina platform (Novaseq 6000 sequencing) according to the standard protocol. 

For data analysis, we refer to the study of Kujawska [33]. Firstly, we performed paired-end reads assembly and quality control: reads were assigned to samples based on their unique barcode and truncated by cutting off the barcode and primer sequence. Then FLASH (V1.2.7) was used to merge paired-end reads and generate raw tags [34]. Next the raw tags were quality filtered under specific filtering conditions to obtain high-quality clean tags [35], according to the QIIME (V1.9.1) [36] quality control process. Finally, the tags were compared with the SILVA database (v132) (16S rRNA metabarcoding data) [37] and the UNITE database (v8.0) (ITS metabarcoding data) [38], using the UCHIME algorithm [39], to detect and remove [40] chimera sequences, and obtain the effective tags.

Representative operational taxonomic unit (OTU) sequences were analyzed using the UPARSE software (v7.0.1001) [41]; the sequences with ≥97% similarity were assigned to the same OTUs, and the bacterial and fungal taxa were assigned based on the SILVA (v132) and the UNITE (v8.0) databases, as mentioned above, with a confidence threshold value of 0.8. Further, alpha diversity indices such as the observed species richness (Sobs), Chao1, and Shannon indices were used to assess the complexity of species in the samples; these indices were calculated using the abovementioned QIIME software (v1.9.1) and displayed using the R software (v2.15.3). Further, a non-metric multidimensional scaling (NMDS) based on the Bray–Curtis distance was performed to determine the similarity of samples in the R project vegan package (v2.6-2; http://CRAN.R-project.org/package=vegan; accessed on 20 April 2022) and the ggplot2 package (v3.3.3; http://CRAN.R-project.org/package=ggplot2; accessed on 1 March 2022). In addition, the OTUs among the different groups were compared by Venn analysis performed using the VennDiagram package (v1.6.16) in R [42]. All these analyses were conducted using the OTU numbers without any model transformation.

#### 2.2.2. Widely Targeted Metabolome Analysis

A widely targeted metabolome analysis based on UHPLC–ESI–MS/MS was performed to identify the metabolite differences between the LC of the DARK and OUT zones at the Metware Biotechnology Co., Ltd. (Wuhan, China), as described earlier [29]. The root and leaf samples were freeze-dried for 48 h and ground into powder. Approximately 100 mg of the powder was extracted with 70% aqueous methanol (0.6 mL), and the extract was analyzed on a UHPLC–ESI–MS/MS system (UHPLC, Shim-pack UFLC SHIMADZU CBM30A system, Kyoto, Japan; MS, Applied Biosystems 4500 Q TRAP, Framingham, MA, USA). Three biological replicates were maintained for each zone’s leaf and root samples. Meanwhile, all the sample extracts were mixed to prepare the quality control (QC) sample used to test the measurement accuracy after every six samples. 

The qualitative analysis of the primary and secondary mass spectrometry data was performed using a self-built database MWDB (v2.0; Metware Biotechnology Co., Ltd. Wuhan, China) and the publicly available databases, such as MassBank (http://www.massbank.jp (accessed on 1 March 2022)), HMDB (Human Metabolome Database; http://www.hmdb.ca (accessed on 1 March 2022)), and METLIN (http://metlin.scripps.edu/index.php (accessed on 1 March 2022)). Meanwhile, the quantitative analysis of the metabolites was performed using the multiple reaction monitoring mode (MRM) of triple quadrupole mass spectrometry. The MultiQuant software (v3.0.2) was used to access the mass spectrometry files and to integrate and correct the peaks. The area of each chromatographic peak represented the relative content of the metabolite; the mass spectra were integrated and corrected to determine the content of each metabolite in the different samples. Further, the levels of each metabolite in the various samples were compared based on the retention time and peak pattern. 

The raw data were processed using the Analyst 1.6.3 software (AB Sciex, Framingham, MA, USA). The original abundance of metabolites was log-transformed to normalize the data and decrease the variance. Principal component analysis (PCA), cluster analysis, and orthogonal projections to latent structures-discriminant analysis (OPLS-DA) were conducted with the metabolite data in R (http://www.r-project.org/ (accessed on 1 March 2022)) following the previously described methods [29]. Variable importance in projection (VIP) values of all metabolites from the OPLS-DA were extracted using the first component. Finally, the differential metabolites of the pairwise comparisons (Leaf samples of CK and FR zones, Leaf-CK vs. Leaf-FR; Root samples of CK and FR zones; Root-CK vs. Root-FR) were screened based on the following criteria: (i) VIP ≥ 1 (high confidence in pairwise comparisons); (ii) a fold change ≥2 and ≤0.5. Further, Kyoto Encyclopedia of Genes and Genomes (KEGG) annotation and metabolic pathway analysis were performed for the differential metabolites. A hypergeometric test was used to identify the significantly enriched pathways (*p* < 0.05).

#### 2.2.3. Correlation Analysis

Finally, the correlation between metabarcoding and metabolome data was analyzed using OmicShare tools, a free online platform for data analysis (https://www.omicshare.com/tools (accessed on 30 April 2022)). We used the Z-score (zero-mean normalization; z = (x − µ)/σ, where x is the original value, µ is the mean value, and σ is the standard deviation value) of the marker metabolites (peak area units) and the OTU numbers of the top ten fungal and bacterial genera to evaluate the correlation. Then, a heat map was generated using the Pearson correlation coefficients with the correlation heat map tools in OmicShare. 

## 3. Results

### 3.1. Microbial Diversity

#### 3.1.1. Metabarcoding Sequencing

**Figure 2:**
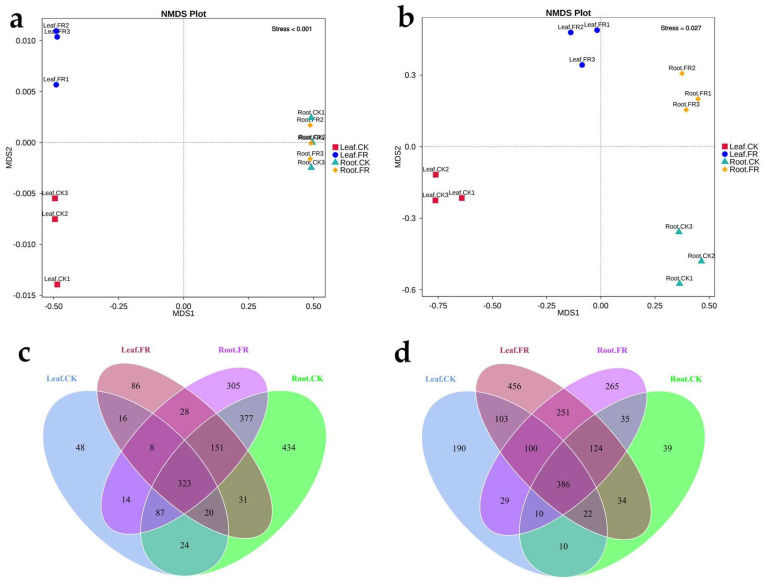
NMDS and Venn analysis of the endophytic bacteria and fungi in the leaves and roots of *Leymus chinensis* (LC) in the DARK (FR) and OUT (CK) zones of *Leucocalocybe mongolica* (LM) fairy rings. NMDS of the (**a**) bacterial and (**b**) fungal OTUs in the FR and CK leaves and roots. Venn analysis of the (**c**) bacterial and (**d**) fungal OTUs.

A total of 2,063,635 effective metabarcoding tags were obtained from 12 samples via sequencing of the 16S rRNA V5–V7 and ITS 1–2 regions. OTU clustering identified 640 bacterial OTUs (16S rDNA) and 665 fungal OTUs (ITS) on average per sample (Table 1). NMDS plots based on the fungal and bacterial OTU abundance in the root and leaf samples showed clear separation (Figure 2a,b), except those of the bacteria in the LC leaves of CK and FR zones that appeared clustered. The Venn diagram based on the OTUs showed 323 and 386 bacterial and fungal OTUs shared among all leaf and root samples (Figure 2c,d). The unique bacterial OTUs (Figure 2c) in the leaves were more in the FR zone (86) than in the CK zone (48), while those in the roots were more in the CK zone than in the FR zone (Root.CK vs. Root.FR, 434 vs. 305). Meanwhile, the unique fungal OTUs (Figure 2d) were more in the leaves and roots of LC in the FR zone (456 in leaf and 265 in root) than in the CK zone (190 in leaf and 39 in root). 

#### 3.1.2. Microbial Alpha Diversity

**Figure 3:**
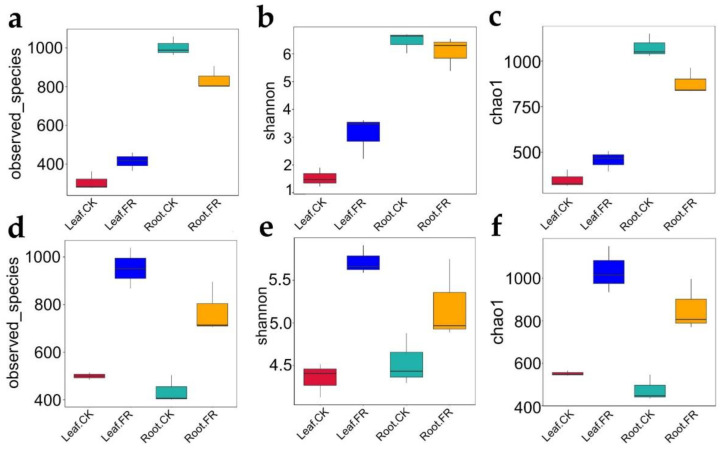
Boxplot showing the alpha diversity indices of the endophytic bacteria and fungi in the LC of FR and CK zones of LM fairy rings. Observed species index of (**a**) bacteria and (**d**) fungi. Shannon index of (**b**) bacteria and (**e**) fungi. Chao1 index of (**c**) bacteria and (**f**) fungi. All indices were calculated based on OTU. The top and bottom whiskers of boxes represent the maximum and minimum values; the line inside the box represents the median, the top margin of the box represents the upper quartile, and the lower margin of the box represents the lower quartile.

We further calculated the alpha diversity indices to determine the species diversity and used the *t*-test to compare the samples. Sobs for the LC leaf and root samples collected from the FR and CK zones ranged from 277 to 1058 for bacteria (Figure 3a) and 485 to 1038 for fungi (Figure 3d). Meanwhile, the Shannon index ranged from 1.22 to 6.7 (Figure 3b) and from 4.12 to 5.74 (Figure 3e), and the Chao1 index from 316 to 1150 (Figure 3c) and from 435 to 1151 (Figure 3f) for bacteria and fungi, respectively. Sobs for bacteria in the leaf samples were significantly higher in the FR zone than in the CK zone (*p* = 0.0053, Figure 3a), while an opposite trend was observed in the roots (*p* = 0.0053, Figure 3a). The bacterial Shannon index in the leaf samples was significantly higher in the FR zone than in the CK zone (*p* = 0.0372, Figure 3b), but no difference was detected between the CK and FR root samples (*p* = 0.2; Figure 3b). The Chao1 index of bacteria in the roots was significantly higher in the CK zone than in the FR zone (*p* = 0.021, Figure 3c), while that in the leaves was not different between the CK zone and FR zone (*p* = 0.0578, Figure 3c). The Sobs values for fungi in the leaves and roots were higher in the FR zone than in the CK zone (Figure 3d; Leaf-CK vs. Leaf-FR, *p* = 0.0017 and Root-CK vs. Root-FR, *p* = 0.0033). The Shannon index values for fungi in the leaf and root samples were also significantly higher in the FR zone than in the CK zone (Figure 3e; Leaf-CK vs. Leaf-FR, *p* = 0.0016 and Root-CK vs. Root-FR, *p* = 0.0175). The Chao1 index for fungi in the leaf and root samples was significantly higher in the FR zone than in the CK zone (Figure 3f; Leaf-CK vs. Leaf-FR, *p* = 0.0013 and Root-CK vs. Root-FR, *p* = 0.0013). These observations confirmed the significant influence of fairy ring ecology on the fungal diversity of LC roots and leaves but a less obvious effect on root bacterial endophytes.

#### 3.1.3. Key Microbial Taxon

**Figure 4:**
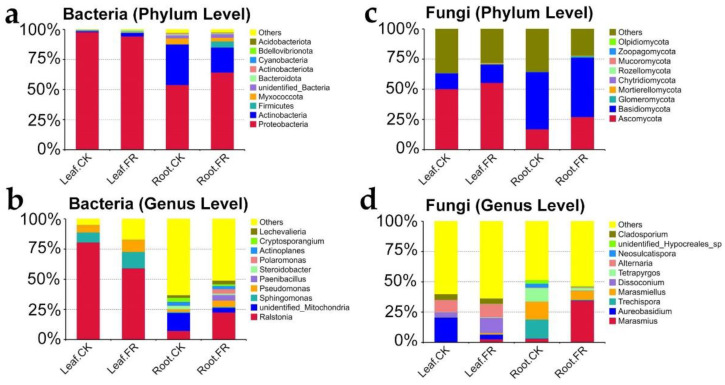
Endophyte community composition and LEfSe analysis of bacteria and fungi in LC of the FR and CK zones of the fairy ring ecosystem. The relative abundance of bacterial community at the (**a**) phylum and (**b**) family levels. The relative abundance of fungal community at the (**c**) phylum and (**d**) family levels.

We further analyzed the differences in the bacterial and fungal communities in the LC leaves and roots between the FR and CK zones based on the SILVA and UNITE databases. The microbes detected are shown in Figure 4. Proteobacteria (97.87%, 94.27%, 53.89%, and 64.28% in Leaf-CK, Leaf-FR, Root-CK, and Root-FR) and Actinobacteria (0.79%, 2.91%, 33.69%, 20.83%) were the top two most abundant bacteria at the phylum level (Figure 4a), and *Ralstonia* (80.57%, 59.15%, 7.24%, 22.56), *Sphingomonas* (8.3%, 13.56%, 0.76%, 0.66%), and *Pseudomonas* (6.03%, 10.0%, 2.18%, 5.34%) were the three most abundant bacteria at the genus level (Figure 4b). Meanwhile, Ascomycota (50.22%, 55.41%, 16.93%, 27.09%) and Basidiomycota (13.09%, 15.14%, 47.4%, 49.27%) were the top two most abundant fungi at the phylum level (Figure 4c), and *Marasmius* (0.44%, 2.62%, 3.13%, 34.45%), *Aureobasidium* (20.23%, 3.89%, 0.12%, 0.22%), *Trechispora* (15.84%, 0.16%, 15.76%, 0.57%), *Marasmiellus* (0.25%, 1.22%, 14.68%, 7.43%), and *Alternaria* (9.99%, 10.86%, 0.33%, 0.72%) were the five crucial fungi at the genus level (Figure 4d). This grouping found that phylum Proteobacteria was dominant in all samples, while the genus *Ralstonia* was the dominant endophytic bacteria in the leaves of all zones. Meanwhile, Ascomycota and Basidiomycota were the dominant fungal phyla, with distribution advantages in both leaves and roots. In addition, the analysis revealed that the bacteria *Sphingomonas* and the fungi *Dissoconium* were enriched in the leaves, while the fungi *Marasmius* was significantly enriched in the roots of LC in the fairy ring ecosystem.

### 3.2. Metabolome Analysis

#### 3.2.1. Metabolite Composition

**Figure 5:**
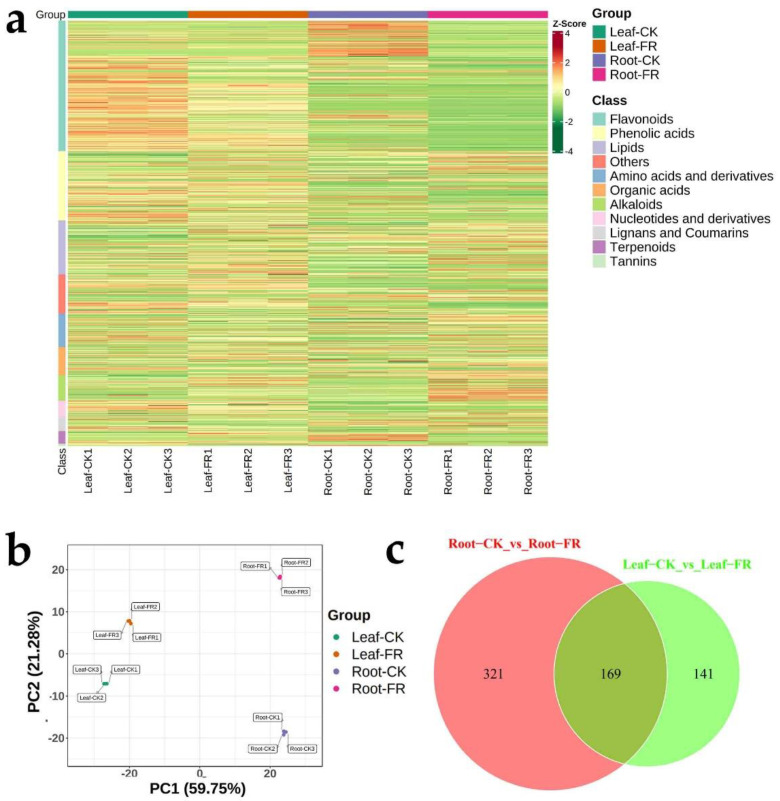
Metabolite composition of LC leaves and roots in the FR and CK zones of LM fairy rings. (**a**) Clustering heat map of all metabolites. Each column represents a sample, and each row represents a metabolite class. A bar with a specific color represents the abundance of each metabolite. Different shades of red and green represent the upregulated and downregulated metabolites, respectively. (**b**) PCA score plot. PC1 represents the first principal component, and PC2 represents the second principal component. The percentage represents the interpretation rate of the principal component of the dataset. A dot represents each sample, and samples indicated in the same color are of the same group. (**c**) The Venn diagram shows the overlapping and unique metabolites among the comparison groups. In the figure, each circle represents a comparison group, and the numbers in the circles and overlaps represent the number of differential metabolites shared between the comparison groups, while the numbers without overlaps represent the number of differential metabolites unique to the comparison group.

We performed a widely targeted metabolomic analysis based on UPLC–ESI–MS/MS approach to reveal the metabolic differences in the LC leaves and roots between the DARK and OUT zones. We evaluated the categories and abundance of metabolites based on Z-scores. A total of 1011 metabolites (grouped into 11 classes), including amino acids and derivatives (79 of 1011), phenolic acids (164), nucleotides and derivatives (38), flavonoids (311), lignans and coumarins (34), tannins (5), alkaloids (61), terpenoids (30), organic acids (67), lipids (129), and others (93), were detected in the leaves and roots (Figure 5a). Detailed information on the metabolites is presented in Supplementary Table S1. The PCA plot (Figure 5b) showed that the samples in the FR zone were significantly different from those in the CK zone, and leaf samples were distinct from the root samples; the groups Leaf-CK, Leaf-FR, Root-CK, and Root-FR were clearly separated. Venn analysis (Figure 5c) showed the differential metabolites shared between Root-CK vs. Root-FR and Leaf-CK vs. Leaf-FR; 169 differential metabolites were common among the leaves and roots in the fairy ring ecosystem. Meanwhile, 321 differential metabolites identified between Root-CK and Root-FR groups were unique to the roots, and 141 differential metabolites identified between Leaf-CK and Leaf-FR were unique to the leaves. Preliminary analysis showed that the fairy rings significantly influenced metabolite synthesis in LC, with a significant downregulation of the flavonoids in roots and leaves of LC in the FR zone (Figure 5a). 

#### 3.2.2. Differential Metabolites

**Figure 6:**
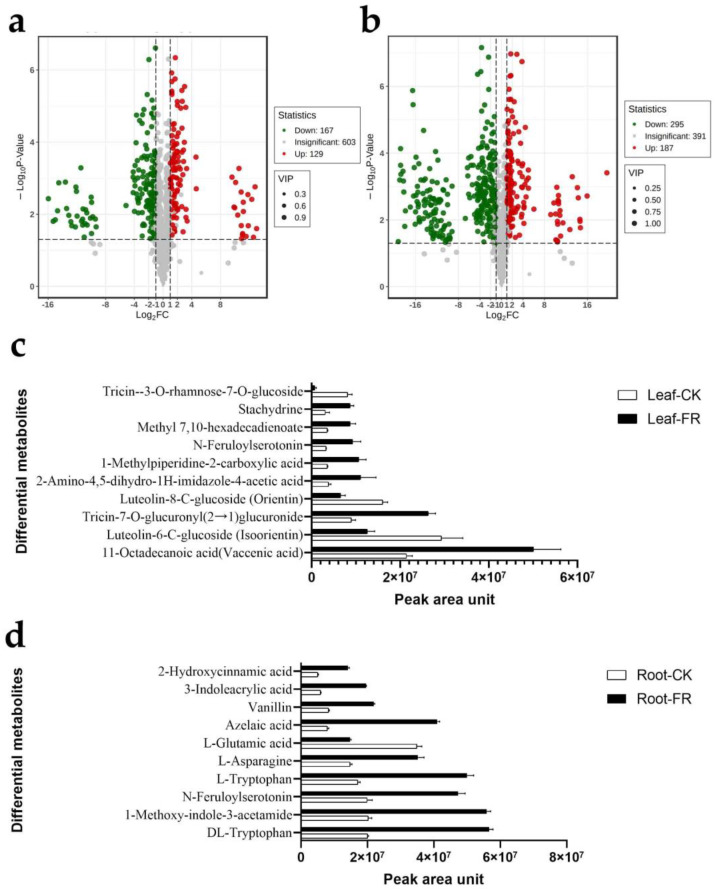
Differential metabolites in LC of FR and CK zones of LM fairy rings. Volcano map of differential metabolites identified from the (**a**) leaves and (**b**) roots of LC in FR and CK zones. Each point in the volcano plot represents a metabolite; green represents the downregulated metabolites, red represents the upregulated metabolites, and gray represents the detected metabolites with no significant difference. The horizontal coordinate represents the log value of the metabolite difference between the FR and CK groups (log_2_FC); the ordinate represents the significance level (−log_10_P-value); the dot size represents the VIP value. Top ten most abundant differential metabolites in (**c**) leaves and (**d**) roots of LC in FR and CK zones. The abscissa represents peak area units, and the ordinate represents the differential metabolites.

Combining the peak area ratio in CK and FR zones, we compared the metabolite abundance among the groups and found 129 metabolites upregulated in leaves (Figure 6a) and 187 in roots (Figure 6b). Then, we identified the top 10 metabolites enriched in the roots and leaves based on the peak area. The top ten metabolites in the leaves were 11-octadecanoic acid (vaccenic acid), luteolin-6-C-glucoside (isoorientin), tricin-7-O-glucuronyl(2→1)glucuronide, luteolin-8-C-glucoside (orientin), 2-amino-4,5-dihydro-1H-imidazole-4-acetic acid, 1-methylpiperidine-2-carboxylic acid, N-feruloyl serotonin methyl, 7,10-hexadecadienoate, stachydrine, tricin--3-O-rhamnose-7-O-glucoside, of which seven were enriched in the DARK zone (Leaf-FR group) and three in the OUT zone (Leaf-CK) (Figure 6c). In roots, the top ten metabolites were DL-tryptophan, 1-methoxy-indole-3-acetamide, N-feruloylserotonin, L-tryptophan, L-asparagine, L-glutamic acid, azelaic acid, vanillin, 3-indoleacrylic acid, and 2-hydroxycinnamic acid, of which all except L-tryptophan were enriched in the DARK zone (Root-FR) (Figure 6d). 

#### 3.2.3. Metabolite Pathway Analysis

**Figure 7:**
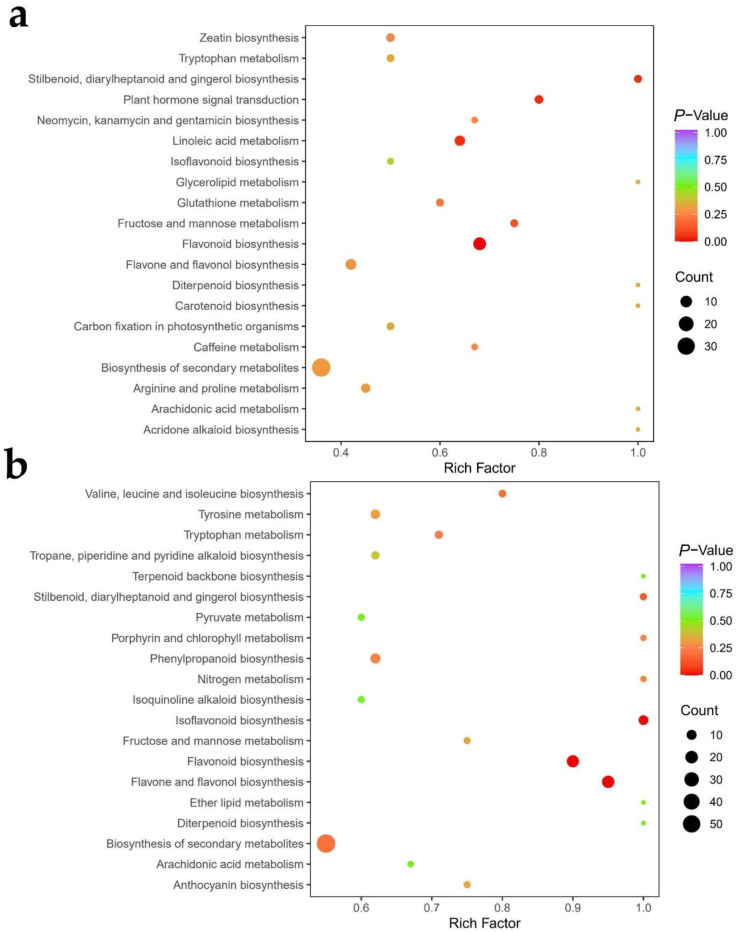
KEGG enrichment analysis of differential metabolites between the CK and FR comparison groups (Leaf-CK vs. Leaf-FR, Root-CK vs. Root-FR). (**a**) represent leaves and (**b**) represent roots. Each bubble in the plot represents a metabolic pathway; the abscissa and the bubble size jointly indicate the magnitude of impact. A larger bubble shows more metabolites are enriched; the bubble color represents the *p*-value of the enrichment analysis, with darker colors indicating a higher degree of enrichment.

We further performed a KEGG enrichment analysis to determine the function of the differential metabolites in a fairy ring ecosystem (Figure 7). In the leaves, significant differences were detected in flavonoid biosynthesis (ko00941), stilbenoid, diarylheptanoid, and gingerol biosynthesis (ko00945), linoleic acid metabolism (ko00591), and plant hormone signal transduction (ko04075) between the CK and FR zones (Figure 7a). Meanwhile, in roots (Figure 7b), flavone and flavonol biosynthesis (ko00944), flavonoid biosynthesis (ko00941), and isoflavonoid biosynthesis (ko00943) showed significant differences between the CK and FR zones. 

**Figure 8:**
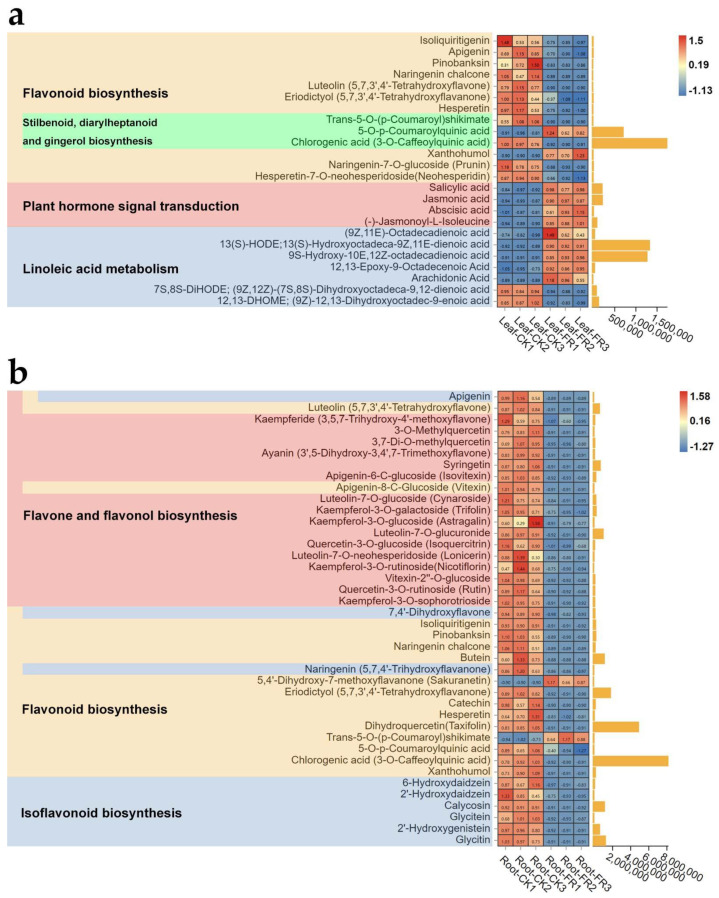
Heat map and histogram showing the KEGG pathways significantly enriched by the differential metabolites in the (**a**) leaf and (**b**) root CK and FR comparison groups (Leaf-CK vs. Leaf-FR, Root-CK vs. Root-FR) of the fairy rings. In the heat map, the abscissa shows the leaf and root samples, and the ordinate represents the metabolites; the color of each grid represents the abundance of metabolites in the sample (normalized by Z-score). In the histogram, the abscissa represents the unit size of the corresponding peak area units; the higher the column, the greater the difference. The pathway to which the metabolite belongs is shown on the left, and the metabolite with multiple colors participates in various pathways. E.g., red means flavone and flavanol biosynthesis pathway.

In leaves, isoliquiritigenin, apigenin, pinobanksin, naringenin chalcone, luteolin (5,7,3′,4′-tetrahydroxyflavone), eriodictyol (5,7,3′,4′-tetrahydroxyflavanone), hesperetin, trans-5-O-(p-coumaroyl)shikimate, chlorogenic acid (3-O-caffeoylquinic acid), naringenin-7-O-glucoside (prunin), and hesperetin-7-O-neohesperidoside (neohesperidin) of the flavonoid biosynthesis pathway (ko00941) were more highly enriched in the CK zone (Leaf-CK) than in the FR zone (Leaf-FR), while 5-O-p-coumaroylquinic acid and xanthohumol were highly enriched in the FR zone (Figure 8a). Meanwhile, 5-O-p-coumaroylquinic acid of the stilbenoid, diarylheptanoid and gingerol biosynthesis (ko00945) was highly enriched in the FR zone while trans-5-O-(p-coumaroyl)shikimate and chlorogenic acid (3-O-caffeoylquinic acid) showed an opposite trend; this pathway is a subpathway of ko00941. Salicylic acid, jasmonic acid, abscisic acid, and (-)-jasmonoyl-L-isoleucine of the plant hormone signal transduction pathway (ko04075) were highly enriched in the FR zone. In the linoleic acid metabolism pathway (ko00591), 9Z,11E)-octadecadienoic acid, 13(S)-HODE;13(S)-hydroxyoctadeca-9Z,11E-dienoic acid, 9S-hydroxy-10E,12Z-octadecadienoic acid, 12,13-epoxy-9-octadecenoic acid, and arachidonic acid were highly enriched in the FR zone while 7S,8S-diHODE; (9Z,12Z)-(7S,8S)-dihydroxyoctadeca-9,12-dienoic acid, and (9Z)-12,13-dihydroxyoctadec-9-enoic acid (12,13-DHOME) showed an opposite trend. In roots, apigenin, luteolin (5,7,3′,4′-tetrahydroxyflavone), kaempferide (3,5,7-trihydroxy-4′-methoxyflavone), 3-O-methylquercetin, 3,7-di-O-methylquercetin, ayanin (3’,5-dihydroxy-3,4′,7-trimethoxyflavone), syringetin, apigenin-6-C-glucoside (isovitexin), apigenin-8-C-glucoside (vitexin), luteolin-7-O-glucoside (cynaroside), kaempferol-3-O-galactoside (trifolin), kaempferol-3-O-glucoside (astragalin), luteolin-7-O-glucuronide, quercetin-3-O-glucoside (isoquercitrin), luteolin-7-O-neohesperidoside (lonicerin), kaempferol-3-O-rutinoside (nicotiflorin), vitexin-2′′-O-glucoside, quercetin-3-O-rutinoside (rutin), and kaempferol-3-O-sophorotrioside of the flavone and flavonol biosynthesis pathway (ko00944) were highly enriched in the FR zone (Figure 8b). The metabolites 7,4′-dihydroxyflavone, isoliquiritigenin, apigenin, pinobanksin, naringenin chalcone, butein, naringenin (5,7,4′-trihydroxyflavanone), luteolin (5,7,3′,4′-tetrahydroxyflavone), eriodictyol (5,7,3′,4′-tetrahydroxyflavanone), catechin, hesperetin, dihydroquercetin (taxifolin), 5-O-p-coumaroylquinic acid, chlorogenic acid (3-O-caffeoylquinic acid), xanthohumol, and apigenin-8-C-glucoside (vitexin) of the flavonoid biosynthesis (ko00941) were highly enriched in the DARK zone, while 5,4′-dihydroxy-7-methoxyflavanone (sakuranetin) and trans-5-O-(p-coumaroyl)shikimate showed an opposite trend. In the isoflavonoid biosynthesis pathway (ko00943), 7,4′-dihydroxyflavone, 6-hydroxydaidzein, 2′-hydroxydaidzein, apigenin, naringenin (5,7,4′-trihydroxyflavanone), calycosin, glycitein, 2′-hydroxygenistein, and lycitin were highly enriched in the roots of the DARK zone. In addition, 5-O-p-coumaroylquinic acid, chlorogenic acid (3-O-caffeoylquinic acid), 13(S)-HODE; 13(S)-hydroxyoctadeca-9Z,11E-dienoic acid, and 9S-hydroxy-10E,12Z-octadecadienoic acid were found accumulated in leaves (peak area units > 500,000), while dihydroquercetin (taxifolin) and chlorogenic acid (3-O-caffeoylquinic acid) were found accumulated in the roots (peak area units > 2,000,000). These observations collectively indicate that the fairy rings significantly reduced the flavonoid synthesis of LC in the FR zone, especially in the roots (Figure 8b). At the same time, they significantly increased plant hormone signal transduction and linoleic acid metabolism in the leaves.

### 3.3. Correlation Analysis

**Figure 9:**
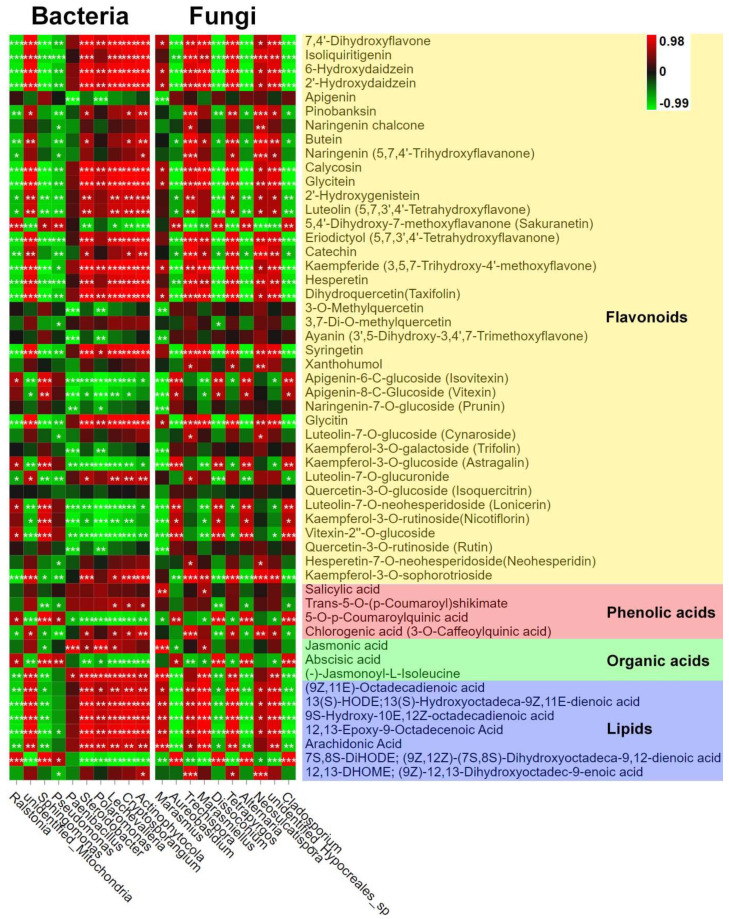
Pearson correlation of top 10 endophytes (bacteria and fungi) at the genus level with the significantly regulated metabolites of seven KEGG pathways. The abscissa represents the endophyte genera, and the ordinate represents the differential metabolites of the seven KEGG pathways. Red and green represent Pearson correlation (r); the key is shown at the top right corner. ‘*’, ‘**’, and ‘***’ indicate significant correlation at *p* ≤ 0.001, 0.001 < *p* < 0.01, and 0.01 < *p* < 0.05; *p* ≥ 0.05 (insignificant): not marked.

Finally, we analyzed the correlation between the endophytes and metabolites of LC based on Z-score normalization. We used the top 10 fungal and bacterial genera with high relative abundance based on OTU numbers to analyze the association with the significantly regulated metabolites (base peak area unit) of the seven pathways identified in Section 3.2.3. We found that the most dominant bacterial genus, *Ralstonia*, negatively correlated with the synthesis of most metabolites; however, one metabolite each of the flavonoid and lipid classes (5,4′-dihydroxy-7-methoxyflavanone (sakuranetin) and 7S,8S-DiHODE; (9Z,12Z)-(7S,8S)-dihydroxyoctadeca-9,12-dienoic acid) showed an opposite trend (Figure 9); the most abundant fungal genus, *Marasmius*, positively correlated with two metabolites of the plant hormone signal transduction pathway (salicylic acid and jasmonic acid) and most lipid class metabolites. Other bacterial and fungal genera were divided into two types based on their association with flavonoids and lipid metabolites. The first type included Steroidobacter, Polaromonas, Lechevalieria, Cryptosporangium, Actinophytocola bacterial genera, and *Trechispora*, *Marasmiellus*, *Tetrapyrgos*, *Neosulcatispora*, and unidentified_*Hypocreales*_sp fungal genera that positively correlated with the flavonoids and lipid metabolites (e.g., 7,4′-dihydroxyflavone and isoliquiritigenin). The second type included *Sphingomonas* and *Pseudomonas* bacterial genera and *Aureobasidium*, *Dissoconium*, *Alternaria*, and *Cladosporium* fungal genera that negatively correlated with flavonoids and lipid metabolites.

## 4. Discussion

Fairy ring ecology generally has the ability to regulate plant growth [8], which is related to the improvement of soil chemical properties [12,18] and secretion of special metabolites by fairy ring fungi [11,43]. In the present study, we focused on how to exploit fairy rings for agricultural use, due to their plant growth promotion ability. LC is one of the main forage grasses in north China, and LM fairy rings are found to inhabit the LC grassland specifically. This ecological phenomenon may be used to improve plant production and has been preliminarily tested (Hu and Wang, personal communication); however, this requires a clear understanding of the factors promoting plant growth. The present study investigated the changes in endophytes and metabolites in LC under an LM fairy ring ecosystem to develop a novel fungus-based fertilizer with substantial application value in agriculture and animal husbandry.

Plants host diverse but taxonomically structured communities of microorganisms; these microbes play crucial roles in regulating plant health [19]. Studies have proven that the non-pathogenic microbes of this category improve nutrient uptake, promote growth, and provide stress tolerance [20]. Therefore, we speculated on the role of such microbes in fairy ring-mediated plant growth promotion and investigated the endophytes of LC in the FR and CK zones. The study detected endophytic fungal and bacterial OTUs in the leaves and roots of LC but at an abundance lower than that detected in the fairy ring soil [18]. In addition, the ratio of endophytic bacteria to fungi detected (0.96:1; 640/665 endophyte diversity) was lower than that reported previously in the soil (2.16:1; 2582/1195 soil microbe diversity). Nevertheless, the endophytic community was more balanced in the plants, with an almost similar proportion of bacteria and fungi, indicating an equal and balanced contribution of fungi and bacteria of the endophytic community as compared to that of the soil community of the fairy ring ecosystem. The number of endophytes in the OUT zone detected in this study is similar to that reported by Zhu from leaves of *Kalidium schrenkianum* (565 bacteria and 606 fungi) [44] and xerophyte shrubs (504 fungi) [45], indicating a stable diversity of endophytes across plants. Venn analysis (Figure 2d) and alpha diversity indexes (Figure 3) showed that the fairy ring ecosystem significantly improved the diversity of endophytic fungi in the leaves and roots of LC in the FR zone, consistent with our previous report on soil fungi as the main factor regulating plant growth in the fairy ring ecosystem [18]. 

The microbial taxonomic analysis identified *Ralstonia* as the most abundant among bacteria and *Marasmius* as the most abundant among fungi in the roots and leaves of the FR zone, which probably played significant roles in promoting plant growth. *Ralstonia* with robust endogenous capacity was present in 50% of all leaf samples and at a high abundance in the roots of LC in the FR zone. Studies have confirmed *Ralstonia* as a harmful bacteria known to cause widespread wilt disease in plants [46,47]. However, LC leaves with abundant *Ralstonia* (Figure 4b) showed no wilt symptoms, which indicated that the *Ralstonia* lacked pathogenicity but had strong infectivity in LC, a result required to be confirmed in future specific assays. This observation suggests the use of the species for developing plant vectors in molecular biology, because through gene editing some microbes can be engineered and contribute to microbial diseases control of plants [48]. Therefore, our discovery of low-virulence pathogens (*Ralstonia*) may provide novel receptor material for potential manipulation in the control of widespread wilt disease.

In addition, the study found an enrichment of *Marasmius*, a widely distributed fairy ring fungus with strong adaptability [5,49], in the roots of LC in the FR zone, suggesting the coexistence of two fairy ring fungi (*Marasmius* and LM). Interestingly, *Marasmius* was enriched in the soil of the FR zone of another LM fairy ring near the sampling site with significant plant growth-promoting characteristics [10]. These observations confirm the coexistence of two fungi in the fairy ring ecosystem, which is a rare phenomenon. Under such a scenario, the two species may compete, and the more adaptable fungi (e.g., *Marasmius*) could weed out the less adaptable one (LM) [7] (this was not observed in the present study) or they might show an additive effect. We might speculate that *Marasmius* in LC also contributed to growth promotion in the LM fairy ring ecosystem, a finding that needs further confirmation. The analysis also detected enrichment of a few other endophytic bacteria and fungi, such as *Sphingomonas* and *Dissoconium*, in the leaves of the FR zone; these microbes might also have contributed to plant growth promotion (Figure 4b,d). Although their abundance was lower than that of *Ralstonia* and *Marasmius*, these results provide a basis for future investigation of the endophytes with growth-promoting abilities in the fairy ring ecosystem. 

We further found that the soil ecological factors and endophytes regulated the metabolism in leaves and roots of LC via the widely targeted metabolome and correlation analysis. We detected more than 1000 metabolites in the LC leaves and roots. Among the top 10 metabolites enriched in the roots and leaves (Figure 9), most showed upregulation in the FR zone, suggesting the role of the fairy ring ecology in improving plant production. Detailed analysis showed that fairy rings significantly reduced the synthesis of flavonoids in the FR zone (Figure 5a). Flavonoids are natural polyphenols abundant in many plants and play essential roles in biological processes and responses to environmental factors [50]. A downregulation of flavonoids is often associated with plant growth inhibition [51], which is opposite to our findings. However, as the fairy ring promoted LC growth and increased biomass, the extra plant size might have diluted the flavonoid concentration; furthermore, the downregulation of flavonoids in the FR zone might be an energy-saving mechanism of LC. Then, due to the stimulation of the synthesis of salicylic acid and jasmonic acid of LC by the ecology of fairy ring, its stress resistance might have been improved, thus reducing the need to synthesize large amounts of flavonoids to enhance resistance. The enrichment of *Ralstonia* in the roots of the FR zone also might have affected the process. Our correlation analysis found a negative association between *Ralstonia* and many flavonoids (Figure 9), consistent with Zhao’s reports [52]. However, the correlation between flavonoid reduction and LC growth promotion in the FR zone needs to be explored. Meanwhile, salicylic acid and jasmonic acid were found enriched in the leaves of LC in the FR zone. Generally, salicylic acid activates resistance against biotrophic pathogens, while jasmonic acid activates defense against herbivorous insects and necrotrophic pathogens [53,54,55]. The endogenous fungi *Paecilomyces variotii* stimulated salicylic acid and jasmonic acid synthesis and improved immunity in Arabidopsis and tobacco [56,57]. Thus, a high level of these phytohormones in the FR zone suggests that the fairy ring improved LC resistance by stimulating salicylic acid and jasmonic acid synthesis. Further, our correlation analysis (Figure 9) indicated an association between salicylic acid and jasmonic acid synthesis in LC and *Marasmius* enrichment (Figure 9). Studies have shown that beneficial endophytes of LC stimulate salicylic acid synthesis and promote pathogen resistance [22]. Thus, the fairy ring ecosystem of LM influenced the distribution of the *Marasmius* genus, which probably stimulated jasmonic acid and salicylic acid syntheses and promoted LC growth.

**Figure 10:**
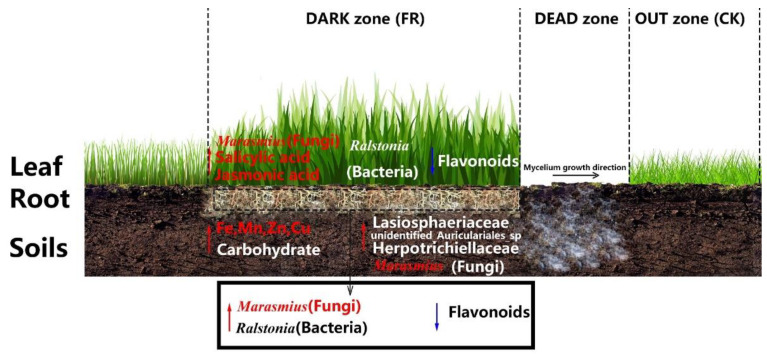
Proposed screening model showing the major factors influencing LC growth promotion in LM fairy rings. The red arrows represent upregulated factors, while the blue arrows represent downregulated factors. Red-labeled elements in the FR zone are important regulatory factors with known functions, and the white-labeled elements are the regulatory factors with unknown ecological functions.

Thus, based on our previous study and the present study’s findings, we propose factors to screen the LM fairy ring that promote LC growth (Figure 10). Our findings collectively indicate that in the LM fairy ring ecosystem, the endophytic fungi *Marasmius* and the metabolites salicylic acid and jasmonic acid were upregulated in the leaves, significantly influencing stress resistance and growth promotion of LC. Meanwhile, the endophytic bacteria of the genus *Ralstonia* were upregulated, and flavonoids were downregulated in leaves and roots but with an unclear role. These observations propose *Marasmius* (upregulation in FR zone), *Ralstonia* (upregulation in FR zone), and flavonoids (downregulation in FR zone) as important factors regulating the ecosystem. In the soil [18], iron, manganese, zinc, and copper ions and the *Marasmius* genus influenced the FR zone; the fungal families Lasiosphaeriaceae, unidentified_Auriculariales_sp, and Herpotrichiellaceae and carbohydrates demonstrated significant influence but with an unclear role. All factors, except the *Marasmius* genus, showed upregulation in the FR soil.

## 5. Conclusions

The present article shows that the fairy ring ecosystem regulates endophytic diversity and metabolic processes and subsequently promotes the growth of LC. Identification of the metabolites of LC based on a widely targeted metabolomic approach provides a reference for improving LC growth under an agricultural system. Finally, we propose factors based on soil analysis in a previous study and the plant root and leaf analysis in this study for screening fairy rings with plant growth-promoting ability. The study improves our understanding of fairy rings and provides novel insights for improving plant production via fairy ring-based management. However, further studies are required to determine the role of flavonoid downregulation, *Marasmius* enrichment, and *Ralstonia* abundance in promoting plant growth in a fairy ring ecosystem.

## Figures and Tables

**Table 1:** Bacterial and fungal metabarcoding sequencing data.

Sample ID	Bacteria			Fungi		
	Clean Tags	Average Length (nt)	OTU Number	Clean Tags	Average Length (nt)	OTU Number
Leaf.CK1	104,742	374	362	84,184	233	485
Leaf.CK2	80,975	374	277	81,380	234	515
Leaf.CK3	98,878	374	283	84,101	239	500
Leaf.FR1	95,560	374	365	74,920	237	1038
Leaf.FR2	96,290	375	418	77,076	235	951
Leaf.FR3	91,267	375	459	82,657	237	868
Root.CK1	90,050	382	964	85,607	267	402
Root.CK2	92,448	384	1058	76,933	261	407
Root.CK3	95,095	392	988	79,489	264	504
Root.FR1	77,247	381	906	75,696	279	706
Root.FR2	85,075	379	803	68,124	267	895
Root.FR3	97,973	378	803	87,868	272	714

## Data Availability

The raw amplicon sequencing dataset of metabarcoding is available in the NCBI Sequence Read Archive under BioSample accession PRJNA853874.

## Supplementary Materials

The following supporting information can be downloaded at: https://www.mdpi.com/article/10.3390/jof8090944/s1, Table S1. Metabolite statistics of leaves and roots of LC.
